# Functional and Structural Plasticity Co-express in a Left Premotor Region During Early Bimanual Skill Learning

**DOI:** 10.3389/fnhum.2020.00310

**Published:** 2020-08-14

**Authors:** Friederike Irmen, Anke Ninija Karabanov, Sophie Alida Bögemann, Kasper Winther Andersen, Kristoffer Hougaard Madsen, Thue Bisgaard, Tim B. Dyrby, Hartwig Roman Siebner

**Affiliations:** ^1^Danish Research Centre for Magnetic Resonance, Centre for Functional and Diagnostic Imaging and Research, Copenhagen University Hospital Hvidovre, Hvidovre, Denmark; ^2^Department of Neurology, Charité—Universitätsmedizin Berlin, corporate member of Freie Universität Berlin, Humboldt-Universität Zu Berlin, Berlin Institute of Health, Berlin, Germany; ^3^Department of Nutrition, Exercise, and Sports, Faculty of Science, University of Copenhagen, Copenhagen, Denmark; ^4^Department of Cognitive Neuroscience, Donders Institute for Brain, Cognition and Behaviour, Radboud University Medical Center, Nijmegen, Netherlands; ^5^Department of Applied Mathematics and Computer Science, Technical University of Denmark, Kongens Lyngby, Denmark; ^6^Surgical Department, Centre for Surgical Science, Zealand University Hospital Køge, Køge, Denmark; ^7^Department of Neurology, Copenhagen University Hospital Bispebjerg, Copenhagen, Denmark; ^8^Institute for Clinical Medicine, Faculty of Medical and Health Sciences, University of Copenhagen, Copenhagen, Denmark

**Keywords:** motor learning, grasping network, endoscopy, sensorimotor, plasticity

## Abstract

**Introduction**: Motor skill learning already triggers the functional reorganization of regional brain activity after short periods of training. Recent studies suggest that microstructural change may emerge at similar timescales, but the spatiotemporal profiles of functional and structural plasticity have rarely been traced in parallel. Recently, we demonstrated that 5 days of endoscopic skill training induces changes in task-related brain activity in the ventral premotor cortex (PMv) and other areas of the frontoparietal grasping network. Here, we analyzed microstructural data, collected during the same experiment to investigate if microstructural plasticity overlaps temporally and spatially with the training-induced changes in task-related brain activity.

**Materials and Methods**: Thirty-nine students were divided into a full-routine group (*n* = 20), that underwent three endoscopy training sessions in the MR-scanner as well as a 5-day virtual reality (VR)-endoscopy training and a brief-routine group (*n* = 19), that only performed the in-scanner endoscopy training sessions. Diffusion Tensor Imaging (DTI)-derived fractional anisotropy (FA) and resting-state functional magnetic resonance imaging (rs-fMRI) were collected at baseline, after the first and after the last VR-training session.

**Results**: The full-routine group showed significant FA changes in a left-hemispheric subcortical cluster underlying the PMv region, for which we previously demonstrated functional plasticity during endoscopy training in the same sample. Functional (task-related fMRI) and structural (FA) changes showed the largest change from the first to the second scan, suggesting similar temporal dynamics. In the full-routine group, the FA change in the subcortical cluster underlying the left PMv scaled positively with the individual improvement in endoscopic surgery.

**Conclusion**: Microstructural white-matter plasticity mirrors the spatiotemporal profile of task-dependent plasticity during a 5-day course of endoscopy skill training. The observed similarities motivate future research on the interplay between functional and structural plasticity during early skill acquisition.

## Introduction

During sensorimotor skill learning, performance typically improves rapidly at early stages followed by slower incremental improvements during later training (Ungerleider, 2002; Dayan and Cohen, 2011). On a neural level, this trajectory is associated with characteristic changes in brain activity: during early learning, neuronal activity increases in sensorimotor areas, which is typically followed by a “pruning” of activity at later learning stages (Dayan and Cohen, 2011; Ma et al., 2011; Makino et al., 2017).

In a recent study, we demonstrated task-dependent activity increases at early learning stages in the frontoparietal grasping network during bimanual skill learning (Karabanov et al., 2019). The training intervention taught medical students basic endoscopy skills over five consecutive days and included endoscopic surgery performance in the fMRI scanner (3 × 32 min; on Day 0, 1 and 5) as well as endoscopy training on a virtual reality (VR)-simulator (5 × 40 min; on Day 1–5). Participants were divided into a “brief-routine” group who only performed the endoscopy tasks during the fMRI scans and a “full-routine” group who completed the full intervention including VR-simulator training. During the three in-scanner endoscopy sessions both groups showed an activity increase in the *left* frontoparietal grasping pathway but only in the full-routine group, the changes were accompanied by a similar activity increase in the *right* frontoparietal grasping network and a modulation of bilateral premotor connectivity (Karabanov et al., 2019).

Here, we investigated with diffusion MRI (dMRI) and resting-state fMRI (rs-fMRI), whether the previously reported task-related functional changes are accompanied by structural and functional plasticity. This is of particular relevance since recent studies suggest that microstructural markers change during the early phase of skill acquisition. Traditionally, structural plasticity has been reported after long training periods where participants engaged in several weeks of training (Scholz et al., 2009; Sampaio-Baptista et al., 2013) but recent work suggests that it can occur on a much shorter time scale and that oligodendrocyte proliferation and differentiation start within the first hours after motor learning (McKenzie et al., 2014; Xiao et al., 2016; Sampaio-Baptista and Johansen-Berg, 2017). Early structural change is detectable using dMRI as demonstrated by Hofstetter et al. (2013) who identified alterations in microstructural features within 2 h of visuospatial memory training (Hofstetter et al., 2013). Recent work has also demonstrated that learning-related microstructural changes spatially overlap with clusters of functional change and persist for up to 12 h following relatively short training sessions (Brodt et al., 2018). Thus, even in the early phases of skill acquisition, functional *and* structural plasticity can be detected in parallel. Skill acquisition also changes resting-state connectivity (rs-connectivity; Albert et al., 2009; Vahdat et al., 2011; Tung et al., 2013; Gregory et al., 2014) and training periods as short as 20 min are enough to modulate functional frontoparietal connectivity beyond the training period (Karabanov et al., 2012). Despite the obvious conceptual interdependence between task-dependent activity, structural and functional plasticity, literature assessing the spatiotemporal trajectories of structural and functional changes during early skill acquisition in parallel is sparse. One rare example is the study by Brodt et al. (2018), who used a memory encoding task and could demonstrate that microstructural plasticity in the posterior parietal cortex occurs within 1 h after learning and overlapped with memory-related functional brain activity (Brodt et al., 2018). Here, we combine dMRI and rs-fMRI data to trace parallel structural and functional plasticity following sensorimotor training reported. We predicted that structural plasticity following bimanual sensorimotor training would be located in the same frontoparietal network that showed task-dependent changes in activity as reported by Karabanov et al. (2019), and that similar spatiotemporal connectivity patterns would also be observed for rs-fMRI.

## Materials and Methods

### Participants

Of the 50 medical students that were recruited for the study, six subjects had to be excluded due to incomplete dMRI data-sets and another five subjects due to incomplete rs-fMRI datasets. Thus, a total of 39 subjects completed all three rs-fMRI and dMRI scans (Table 1). Participants were randomly assigned to a full-routine [*n* = 20, age *M*(SD) = 24.25(2.07), 45% female] or brief-routine group [*n* = 19, age *M*(SD) = 22.68(2.13), 58% female]. The full-routine group underwent three sessions of in-scanner endoscopy performance and an additional 5-days of training on a VR-simulator. The brief-routine group only performed the in-scanner training. Subjects had no prior experience with endoscopic surgery, no history of psychiatric or neurological diseases, and were not taking any neuroactive medication. The full-routine and brief-routine group did not differ in age (*t*_(1,37)_ = 0.53, *p* = 0.60), hours slept before the experiment on either day [*t*_(1,37)_ = 0.94, *p* = 0.44; average hours of sleep: *M*(SD) = 7.37(1.31) in the full-routine group and *M*(SD) = 7.49(1.28) in the brief routine group], the hours subjects were awake before arriving for the experiment [*t*_(1,37)_ = 0.29, *p* = 0.79; age hours awake: *M*(SD) = 4.13(2.26) in the full-routine group and *M*(SD) = 4.03(1.93) in the brief routine group], or the time they spent playing video games per month (*t*_(1,37)_ = 0.17, *p* = 0.86). In both groups, participants exercised equally regularly [95% in full-routine and 79% in control group; *X*^2^ (1, *N* = 39) = 3.14, *p* > 0.05]. In the full-routine group 55% of subjects played a musical instrument while in the control group it was only 21%; *X*^2^ (1, *N* = 39) = 4.74, *p* = 0.03. Handedness was assessed using the short Edinburgh Handedness Inventory (Oldfield, 1971; % right-handers in the full-routine group: 93; brief-routine group: 82). Subjects received detailed instructions about the experimental procedure, signed informed consent, and were reimbursed for their participation. The study was approved by the Regional Committee on Health Research Ethics of the Capitol Region in Denmark following the declaration of Helsinki. Written informed consent was obtained from the individuals for the publication of any potentially identifiable images or data included in this article.

**Table 1:** Sample demographics.

	Full-routine group	Brief-routine group
N	20	19
Women	9	11
Age *M(SD)*	24.25 (2.07)	22.68 (2.13)
Right handedness (%)	93.42	81.50
Semester studying *M(SD)*	6.35 (3.36)	5.89 (2.89)
Interested in surgical career (%)	35	47
Exercising regularly (%)	95	79
Playing an instrument (%)	55	21
Video game sessions per month *M(SD)*	6.80 (4.81)	5.68 (4.25)
Hours of sleep (average D0, D1, D5) *M(SD)*	7.37 (1.31)	7.49 (1.28)
Hours awake (average D0, D1, D5) *M(SD)*	4.13 (2.26)	4.03 (1.93)

*M(SD), Mean (Standard deviation)*.

### Experimental Procedure

**Figure 1:**
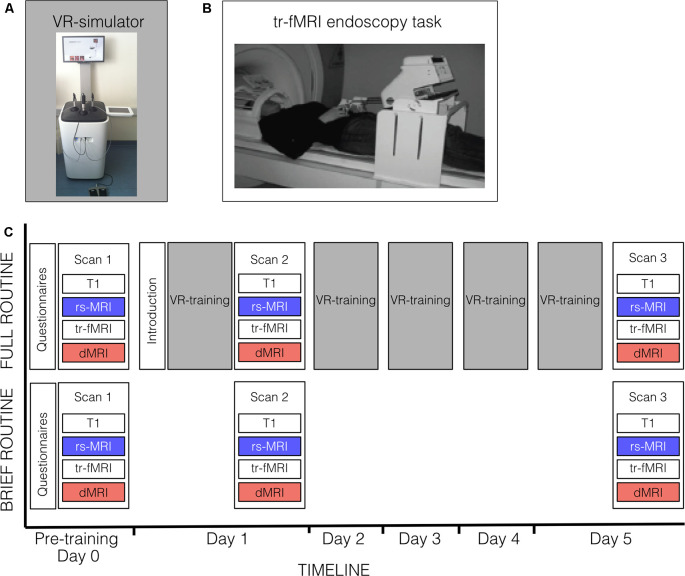
Bimanual skill training and experimental procedure. **(A)** Endoscopic virtual reality (VR)-simulator set-up for bimanual skill training. **(B)** Set-up of in-scanner endoscopy task. Results for task-related activity changes during in-scanner endoscopy training are reported elsewhere (Karabanov et al., 2019). **(C)** Subjects of the full-routine group underwent resting-state fMRI (rs-fMRI), task-related fMRI (tr-fMRI) and diffusion MRI (dMRI) scans before (Scan 1) and after (Scan 2) the first VR-training session (approx. 45 min) on the endoscopic VR-simulator, followed by four consecutive days of VR-training, and received a third scan (Scan 3) after their last VR-training session. A 10-min introduction preceded the first VR-training session. Subjects of the brief-routine group got scanned in the same timeframe without receiving VR-training in endoscopy. Both groups completed a set of questionnaires before their first examination.

Figure 1 provides an overview of the experimental design. All participants were scanned on three occasions (Day 0, Day 1, Day 5) and each scanning occasion included an rs-fMRI session (≈8 min), four blocks of task-related fMRI (tr-fMRI) (≈8 min each), a diffusion-weighted MRI scan (≈26 min) and a T1-weighted anatomical scan (≈6 min). The first scanning session represented a baseline scan (Scan 1; Day 0). On the following day (Day 1), subjects of the full routine group got an introduction to training on the endoscopic VR-simulator (approx. 10 min), underwent their first endoscopic training session (approx. 45 min), and immediately afterward took their second scan (Scan 2). The brief-routine group directly underwent Scan 2 without receiving any training beforehand. For the following four consecutive days of the week (Day 2–Day 5), the full-routine group trained on the VR-simulator each day at a similar time of day for approximately 45 min. On Day 5, after completing their last training session, subjects got their last scan (Scan 3). The brief-routine group also returned on Day 5 for Scan 3. Subjects of both groups completed a set of standardized questionnaires on medical career plans, experience playing musical instruments, and video gaming (Table 1).

### Image Data Acquisition

Data acquisition was performed on a 3T Achieva scanner (Philips, Best, The Netherlands) at the Danish Research Centre for Magnetic Resonance, Hvidovre Hospital, Copenhagen, Denmark. A scanning session consisted of a 3D structural T1-weighted sequence magnetization-prepared rapid acquisition with gradient echo [MPRAGE; repetition time (TR): 5.9 ms, echo time (TE): 2.7 ms; flip angle: 8°, Field-of-View (FOV): 245 mm; 245 slices; resolution 0.85 mm^3^], a whole-brain coverage resting-state T2*-weighted fMRI Echo-planar Imaging (EPI) sequence (FOV: 192 mm; 32 slices; TR/TE: 2,000/30 ms; resolution: 0.23 mm^3^; flip angle: 90°), and three diffusion-weighted MRI SE single-shot EPI sequences (FOV: 224 mm; TR/TE: 10,710/85; image resolution: 2.3 mm^3^, flip angle: 8°). The three-shell dMRI included shells with a *b-value* of 2,000 and 1,000 s/mm^2^ acquired in 62 non-collinear directions, and a *b-value* of 300 s/mm^2^ acquired in six directions, each dMRI shell was initiated with a *b*_0_ i.e., 0 s/mm^2^, scan. For each shell, two reversed phase-encoding *b*_0_ i.e., 0 s/mm^2^, were acquired as reference scans for susceptibility correction using the FSL topup tool (Andersson et al., 2003; Smith et al., 2004; Jenkinson et al., 2012). Additionally, we acquired four task-related T2*-weighted fMRI sequences during simple laparoscopy tasks, that have been reported in detail by Karabanov et al. (2019; EPI; FOV: 192 mm; 32 slices; TR/TE: 2,000/30 ms; resolution: 3 × 3 × 4 mm^3^, flip angle: 90°). Subjects’ scans were scheduled at the same time of day on all three occasions and although there was variance across the group in subjects scanned in the morning and the afternoon, this distribution was kept stable between training and control group.

### In-Scanner Endoscopic Training

Both the brief- and full-routine groups completed three sessions of in-scanner endoscopy training during the tr-fMRI scans (Figure 1B). The tasks included a pointing and a transfer task that respectively trained simply moving the endoscopic instruments (pointing) and using the instruments to pick up small objects and transfer them to the other hand (transfer) as well as rest periods. The performance was quantified by the speed of pointing and transfer movements as well as by the accuracy of transfer movements (for details on the task and analysis see Karabanov et al., 2019).

### Endoscopic VR-Training

The full-routine group completed a 5-day VR-training regime on an endoscopic VR-simulator (Figure 1A; LapSim Haptic System, Surgical Science, Gothenburg, Sweden^1^, for details see Karabanov et al., 2019). In brief, subjects stood in an upright position with a surgical instrument in each hand while monitoring their movements on a screen. VR-training tasks comprised standard endoscopic exercises such as bimanual transfer, grasping, cutting, lifting, and suturing. Task performance was monitored through automatic recording of movement parameters measuring speed, accuracy, and smoothness of movement. We used the same performance metrics as Karabanov et al. (2019): to compute an overall mean performance score for each VR-training session all individual scores of movement speed, smoothness and several accuracy measures for each hand in each task were normalized so that values lay between 0 and 1. This was done by subtracting the smallest value of the particular performance metric in the entire group from each raw value and then dividing it by the metric’s range. Following this normalization, all measures were summed and divided by the total number of measures to create a “session score” combining information about movement smoothness, accuracy, and speed.

### Analysis of dMRI Data

#### Preprocessing

dMRI data were preprocessed using an in-house pipeline written in Matlab (R2013a, version 8.1; The Mathworks, Natwick, MA, USA) based on common FSL tools [FSL version 5.0.2.2; Functional MRI of Brain (FMRIB) Software, Oxford University, Oxford, UK]. It applied a correction for susceptibility artifacts, motion artifacts, and eddy currents, updated the brain mask, linearly co-registered dMRI images to the MNI template (applying the default interpolation method in FSL) and corrected gradient directions for rotations during the alignment of the dMRI data set. The diffusion tensor imaging (DTI) model was fit to the *b* = 1,000 s/mm^2^ dMRI dataset with the weighted linear least squares method (Basser et al., 1994). The DTI model was selected because of its well documented favorable sensitivity profile to detect plastic changes in the microstructural environment. However, a downside of DTI is its limited tissue compartment specificity. On a microstructural level, training-related alterations in DTI parameters could relate to changes in fibers and cell body anisotropy as well as (indirectly) to myelin changes. At the macroscopic level, training-related alterations could describe fiber dispersion in areas with crossing fibers. Certainly, DTI is a simple model when compared to other more specific diffusion models and it cannot correctly model crossing fibers; however, it benefits from being independent of *a-priori* assumptions. Here, we assumed that any training-induced plastic changes would appear at a microstructural level and that they could vary along different tracts. Thus, we concentrated on assessing the DTI parameters Fractional Anisotropy (FA), mean diffusivity (MD) as well radial (RD), and axial (AD) diffusivity (Jones, 2004; Wheeler-Kingshott and Cercignani, 2009).

#### Tract-Based Spatial Statistics (TBSS)

To quantify white matter microstructural plasticity induced by training, we ran a TBSS analysis (Smith et al., 2006). A white matter skeleton template that contained a voxel-to-voxel correspondence across subjects was created as follows: FA maps from each subject for all scans were averaged to create a subject-wise average FA map. The subject-wise FA map was nonlinearly registered to the FMRIB58_FA 1 × 1 × 1 mm MNI-space image and the normalized subject-wise maps of all study subjects were averaged. Finally, the resulting study-average FA map was thresholded to an FA value of 0.3 to obtain a robust white matter skeleton (Smith et al., 2006). To be able to use the skeleton as a mask for the FA maps of each subject for each scan, the individual FA maps of each subject were also non-linearly registered to MNI space. The skeleton was used as a mask to extract the FA values for each scan of each subject, which were then used for voxel-wise comparisons within- and between groups. For quality control, FA maps were visually inspected for distortion artifacts before analysis and the TBSS white matter skeleton was checked to cover only white matter. The analysis was repeated for RD, AD, and MD.

#### Selection of Tracts of Interest (TOI)

In addition to whole-brain TBSS skeleton analyses, we ran analyses restricted to tracts of interest (TOI). Based on our previous findings showing training-dependent activity changes in the frontoparietal grasping network, we selected the bilateral superior longitudinal fasciculus (SLF) as the primary TOI. Besides, we also performed a more exploratory analysis on the bilateral corticospinal tract (CST), and the corpus callosum based on their general relevance during motor learning and interhemispheric transmission (Tuch et al., 2005; Blumenfeld-Katzir et al., 2011; Steele et al., 2012; Sampaio-Baptista et al., 2013; Wang et al., 2016). All TOI were identified by John Hopkins University white matter atlas (JHU-ICBM white-matter labels, Mori et al., 2005).

#### Statistical Analysis of dMRI Data

We applied a General Linear Model (GLM) and randomized permutation testing (*randomise* tool, FSL) with a paired-sample design comparing maps over time for the full-routine and brief-routine group separately. Here, we directly compared whole-brain or within-TOI FA skeletonized maps of separate scanning sessions (Scan 1 vs. Scan 2; Scan 1 vs. Scan 3) within each group. We applied Threshold-Free Cluster Enhancement (TFCE) as a cluster-like voxel-wise statistic (Smith and Nichols, 2009) using the standard parameters for 2D data and tested for significance on a level of *p* < 0.05 after FWE correction as implemented in *randomise*. We visually checked that statistical significance clusters lay within the white matter in all subjects. To allow for a more direct comparison between groups, we supplemented the analysis by a repeated-measures ANOVA in SPM12, where we extracted the FA maps of the TOI for each subject at each scanning occasion and applied a GLM with the factors group (full-routine or brief-routine) and time (Day 0, Day 1, Day 5). To correct for multiple comparisons, voxel-wise FWE correction was applied (*p* < 0.05). To rule out possible baseline differences, we tested whether baseline FA values (at Day 0) were significantly different between the two groups in the TOI.

### Analysis of rs-fMRI Data

#### Preprocessing

rs-fMRI data were analyzed using SPM12 (The Wellcome Trust Centre for Neuroimaging, London, UK). First, both the MPRAGE and the EPI sequences were manually reoriented based on the anterior/posterior commissures. Then, all EPI images were slice time corrected using a temporal center slice as a reference and realigned to the mean EPI image (within every session) to correct for small head movements. The images were normalized to the MNI-template, resampled to 2 mm isotropic voxels and smoothed with an isotropic 6 mm full-width half-maximum Gaussian kernel.

#### Independent Component Analysis

Inter-regional connectivity was determined by a spatial independent component analysis (ICA) using the Group-ICA of fMRI Toolbox (GIFT; Medical Image Analysis Lab, New Mexico, USA). First, the dimensions of the dataset were reduced during a principal component analysis (PCA) on subject-level. Subsequently, the data were temporally concatenated before another PCA was performed. After these two steps of data-reduction, the actual ICA was performed using the InfoMax algorithm with default parameters (Bell and Sejnowski, 1995). Subject-specific spatial maps were estimated using back reconstruction. Based on the results of Karabanov et al. (2019), we selected two ICA templates (Smith et al., 2009) for which we expected to see learning-induced changes: the sensorimotor network (SMN) and the frontoparietal network (FPN). The default mode network (DMN) was included as a third template where changes were not expected to be specific to the training context although unspecific effects based on e.g. increased familiarity with the scanning environment cannot be ruled out.

#### Statistical Analysis of rs-fMRI Data

We compared groups using a flexible factorial GLM applying a repeated-measures design with the factors group (full-routine or brief-routine) and time (Day 0, Day 1, and Day 5) comparing ICA-based subject-specific spatial maps. We assessed the significance of cluster sizes using a random permutation test as implemented in *randomise* with a cluster defining threshold of *p_uncorrected* < 0.001 and considered only voxels within the corresponding templates [SMN, FPN and DMN template (Smith et al., 2009)]. Further, we tested whether baseline values (at Day 0) were significantly different between the two groups in networks with a significant effect.

### Correlation With Behavior

We checked if the behavioral improvements in VR-training in the full-routine group were associated with the observed structural changes. To this end, the performance improvement in the VR-training score (Δ Day 5–Day 1, i.e., from the first day of VR-training to the last day of VR-training) was correlated with the average change in FA (Δ Day 5–Day 0, i.e., from baseline scan before any VR-training to the last day of VR-training). As a *post hoc* analysis, to assess a putative interaction between microstructural white matter changes and rs-connectivity changes, we correlated their average change in the significant clusters. For correlation analyses, we used randomized permutation tests (5,000 permutations) to test for significance (*p*-values are reported at a 5% level after correcting for multiple comparisons) and used Spearman’s correlation coefficients throughout all analyses.

## Results

### Structural Changes in White Matter Tracts

**Figure 2:**
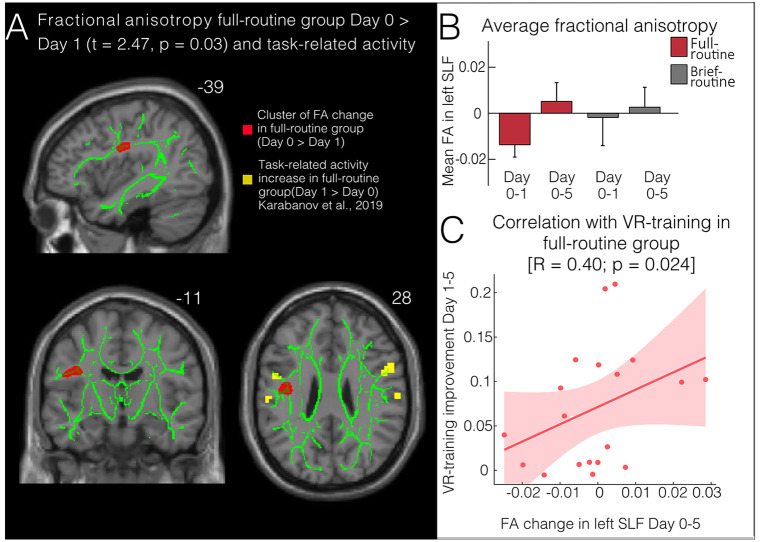
Structural changes following bimanual skill learning. **(A)** Fractional anisotropy (FA) decreases in full-routine subjects (but not brief-routine subjects) after one training session on the VR-simulator in a cluster (red) in the left superior longitudinal fasciculus (SLF) (visualized using tbss_fill). Significant clusters of change in task-related fMRI (yellow; Karabanov et al., 2019) lie very close to the clusters of structural change. Both superimposed with the TBSS skeleton (green). **(B)** Average FA on Day 1 and Day 5 compared to baseline (Day 0). There is a significant change in FA from Day 0 to Day 1 in the full-routine group. **(C)** Improvement of VR-endoscopic skill performance from the first to the last day of training correlates with changes in FA in the left SLF from the first to the last day of training.

For the full-routine group, a significant decrease in FA in the left SLF precentral white matter was detected from Day 0 to Day 1 (58 voxels, peak-level MNI coordinates: *x* = −39, *y* = −11, *z* = 28, *t_max_* = 2.74 and *p_FWEcorr_* = 0.03, Figures 2A,B). This change did not persist when testing Day 0 against Day 5. No change could be observed in the brief-routine group. Both MD and AD also decreased slightly, although not significantly; and there was a trending increase in RD from Day 0 to Day 1 close to the region where FA was significantly changed (34 voxels, peak-level MNI coordinates: *x* = −36, *y* = −12, *z* = 26, *t_max_* = −1.63, *p* = 0.06).

To compare FA changes between groups, subjects’ FA maps within the relevant significant clusters were compared in a GLM with the factors group and time. This analysis revealed that the above-described focal modulation of FA in the precentral white matter of the SLF did not suffice for a significant time by group interaction. The GLM did not show a significant main effect. Additionally, a paired T-test comparing average FA values in the left SLF at baseline did not show a significant group difference (*t* = 0.95, *p* = 0.35), hence we normalized the change in FA to baseline in our visualizations.

### Connectivity Changes in the Sensorimotor Network

**Figure 3:**
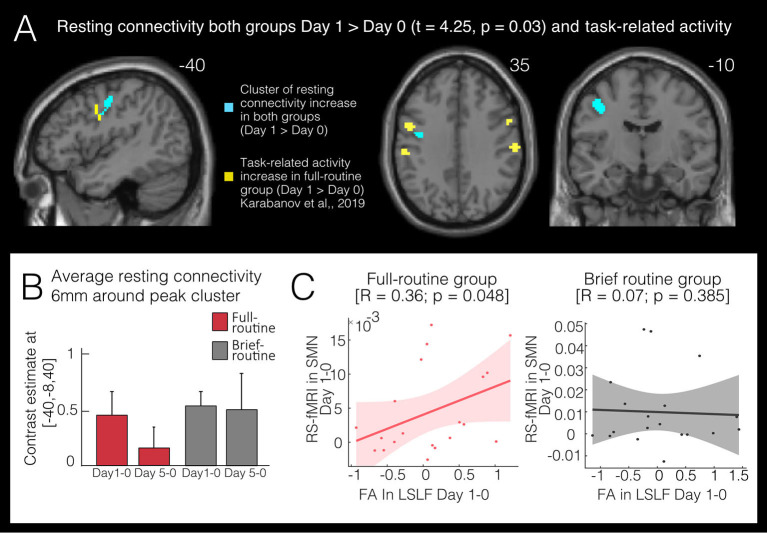
Functional changes following bimanual skill learning. **(A)** Resting-state sensorimotor connectivity increases in both full-routine and brief-routine group in a left premotor cluster after one day of in-scanner training (Day 1 > Day 0). Significant clusters of change in task-related fMRI (yellow; Karabanov et al., 2019) lie in close spatial proximity to the resting-state clusters (blue). **(B)** Average resting-state (rs-)connectivity in both groups in significant clusters (Day 1 > Day 0) within a 6 mm sphere around peak voxel (−40, −8, 40). Both groups show increased rs-connectivity on Day 1 compared to baseline but only the full-routine group showed a correlation **(C)** between the increase in functional rs-connectivity and the decrease in FA (*r* = 0.36, *p_corr_* = 0.05). Also, only the full-routine group showed a tendency of rs-connectivity lowering back to baseline levels at the last scanning session.

For rs-connectivity, 20 components were estimated in the ICA and three components showed sufficient correlations (*r* > 0.25, Smith et al., 2009) with the SMN (*r* = 0.45), the FPN (*r* = 0.60) and the DMN (*r* = 0.70) templates, respectively. In the SMN we found an increase in rs-connectivity in both groups at Day 1 compared to baseline (Figures 3A,B): inter-regional rs-connectivity increased in the left premotor cortex (143 voxels peak cluster: *x* = −40, *y* = −8, *z* = 40) in both the full- and brief-routine group from Day 0 to Day 1, *t_max_* = 4.25 and *p_FWEcorr_* = 0.029. There was no significant main effect of group or interaction of time by the group. The baseline rs-connectivity in a 6 mm sphere around the significant cluster in the SMN did not differ at baseline between groups, *t* = −1.34, *p* = 0.18.

For the FPN no significant main effects or interactions were observed. In the DMN no effect of time nor time by group interaction was observed, but a general difference between groups was indicated in the left precuneus (146 voxels, peak-level MNI coordinates: *x* = −4, *y* = −68, *z* = 28, *t_max_* = 4.59 and *p_FWEcorr_* = 0.049).

### Correlations With Behavioral Parameters

Performance on the VR-simulator in the full-routine group improved steadily over the 5 days (*p* < 0.001, see Karabanov et al., 2019 for more details). VR-training improvement was correlated with change in FA in the significant cluster of the left SLF from Day 0 to Day 5 (*r* = 0.4, *p_corr_* = 0.02, Figure 2C). This indicates that full-routine subjects who showed the largest performance improvement over five training days also had the highest change in FA values. Changes in VR-performance did not correlate with changes in resting-state connectivity. Finally, due to the close spatial proximity of the left premotor cortex, the cluster in which both groups increased in rs-connectivity from Day 0 to Day 1 and the left SLF cluster in which we detected an FA decrease from Day 0 to Day 1 in the full-routine group, we ran a *post hoc* analysis and extracted rs-connectivity and FA values for both groups in these clusters. We correlated the average change in the respective significant clusters and found a correlation between FA and resting-state connectivity change in the full routine group (*R* = 0.36; *p_corr_* = 0.05, Figure 3C), which was absent in the brief routine group (*R* = 0.07, *p_corr_* = 0.38).

## Discussion

We found that the spatiotemporal expression of microstructural plasticity during early bimanual skill learning paralleled task-related functional plasticity induced by endoscopic exercises (Karabanov et al., 2019). In the time domain, functional and structural plasticity showed the greatest changes after the initial training session. In the spatial domain, functional and structural plasticity converged in the left ventral premotor cortex (PMv) and adjacent subcortical white matter. Early bimanual skill learning also led to a training-depended upregulation of rs-connectivity in the left PMv and the magnitude of change in rs-connectivity correlated with the decrease in FA for participants that completed the full training routine. Taken together, our data show that structural and functional connectivity markers converge in the premotor cortex during the learning of a complex bimanual skill. The similarities in the spatiotemporal profiles of functional and structural plasticity markers motivate future work to investigate the interactions between functional and structural plasticity during early skill acquisition.

### Use-Dependent Plasticity in Subcortical White Matter

In the full-routine group, regional FA in the left SLF underlying the PMv region decreased significantly after one day of VR-training. Spatially, the FA change was located in a part of the SLF subjacent to the PMv, a region that also showed the greatest task-related change (Karabanov et al., 2019). An FA *decrease* may initially seem unexpected, as the majority of studies report increases in FA after motor skill training (Scholz et al., 2009; Sampaio-Baptista et al., 2013). However, changes in white matter microstructure can lead to either a decrease or increase in FA depending on crossing macrostructural features (i.e., widespread changes along fiber tracts) and microstructural changes between their volume fractions (Wheeler-Kingshott and Cercignani, 2009; Andersen et al., 2020). DTI metrics like RD and AD link in expressing the composite measure FA and can aid interpretation of FA changes. Our data showed a trend toward increased RD, which may, together with a nominal decrease in AD, account for the significant FA findings. All changes in DTI metrics were observed in the same part of the SLF and only showed sporadic spread along the whole tract, suggesting a local geometrical change in the microstructural environment that was not detectable at other parts of the tract. Thus, the short-term decrease of FA (in correspondence with decreased RD) observed locally in the left SLF most likely indicates a microscopic change in few fiber bundles caused by an increase in regional myelination. Rodent experiments support the notion that use-dependent microstructural changes, such as the formation of myelin and dendritic spines occur as early as 1 h after learning (Xu et al., 2009; Lesburguères et al., 2011; Cowansage et al., 2014; Kitamura et al., 2017) and several studies have suggested that these early white matter changes are driven by expression of myelination-related genes in response to experience and neuronal activity (Sagi et al., 2012; Chorghay et al., 2018; Sampaio-Baptista et al., 2020).

Regional FA changes underlying the PMv did not remain significantly modulated at the last scan suggesting that modulations during early training may depend on different processes than slower-acting plasticity during long-term skill acquisition. This idea is partially supported by the correlation we found between FA change and the skill performance change from the first to the last day of VR-training: FA change was greatest in subjects who showed the strongest overall improvement during VR-training, suggesting that the initial dip in FA during early learning can reverse to increased FA-values and that a long-term increase in FA in the SLF is tied to good skill performance. However, additional research relating short-term and long-term motor learning to FA changes is required to test this hypothesis.

A more exhaustive understanding of local changes in complex white matter regions with many crossing fibers like the SFL might be gained by the exploration of novel parameters such as micro-FA, a metric that is not confounded by the influence of fiber orientation dispersion (Lasič et al., 2014; Szczepankiewicz et al., 2015; Lundell et al., 2019). A recent study by our group (Andersen et al., 2020) compared white matter changes in patients with multiple sclerosis (MS) detected by FA and by micro-FA and found that while FA and micro-FA showed the same decrease with worsening MS pathology in straight, homogeneous white matter regions, the two metrics showed change pointing in opposite directions in complex, heterogenous fiber tracts: while FA increased with progressing disease, micro-FA decreased and this was interpreted as axonal degeneration of crossing fibers that impact FA and micro-FA differentially (Andersen et al., 2020). For future studies, micro-FA could be helpful for the better tracing of training-induced white matter microstructural changes.

### Use-Dependent Plasticity in Resting-State Connectivity

In the left PMv, we were also able to detect an increase in rs-connectivity from the first to the second scan session. While participants in both groups displayed the initial increase, only the full-routine group showed a correlation between the increase in rs-connectivity and the decrease in FA. Moreover, only the full-routine group showed a tendency of rs-connectivity lowering back to baseline levels at the last scanning session. Together, this may suggest that while simple task-exposure is enough to upregulate rs-connectivity, only longer training intervals connect these changes to training-induced plasticity effects. However, the exact interaction underlying FA and rs-connectivity indices requires a more detailed investigation.

Previous research has demonstrated that resting-state networks are influenced by recent experiences (Stevens et al., 2010; Tambini et al., 2010; Kannurpatti and Biswal, 2012; Choi et al., 2015) and that task-related neuronal activity is linearly predicted by the level of resting-state activity (Kannurpatti and Biswal, 2012). However, in our study the longevity of resting-state changes following task-exposure is of note: although resting scan measurements were performed before the in-scanner training, rs-connectivity changes within the PMv were detected 24 h after the last instance of task training. It is however possible that situational cues may have led participants to reactivate network-nodes tagged as relevant during the last in-scanner training session and thereby amplified training-related resting-state effects in task-relevant networks (Lesburguères et al., 2011).

### Short-Term Structural and Functional Plasticity Following Learning

Our data show that early training plays a pivotal role in the physiological response in dMRI and fMRI. The microstructural changes that are the focus of this article only occurred after the first training day and followed the spatiotemporal pattern of training-dependent changes observed during task performance. These findings are coherent with other studies reporting the greatest plasticity changes at the very beginning of training (Brodt et al., 2018; Bönstrup et al., 2019) and such early physiological responses may indicate an initial plasticity overshoot in response to novel task exposure that is followed by more incremental plasticity changes (Molina-Luna et al., 2008; Yotsumoto et al., 2008; Ma et al., 2011; Reed et al., 2011; Wenger et al., 2017). Future studies investigating the time course of early plastic changes at a higher temporal resolution are warranted to better understand the relationship between a potential initial overshoot and later incremental changes.

The here presented data suggest that the left hemispheric grasping pathway is especially reactive to training-induced structural plasticity. Interestingly, these results are coherent with the results of Karabanov et al. (2019), which also reported laterality effects. Specifically, Karabanov et al. (2019) found that left-hemispheric changes in PMv activity occurred already in response to task-repetition in the scanner, i.e. the newly learned bimanual hand-to-tool transformations were associated with changes in the left PMv. For the right hemispheric activity to change further training on the VR-simulator was required. Together, this data suggests the left frontoparietal grasping pathway to be more reactive to the early effects of bimanual skill training. Concurrently, there is evidence for the left-hemispheric laterality of the grasping pathway which is activated during bimanual reach-and-grasp tasks (Serrien et al., 2003; van den Berg et al., 2010; Blinch et al., 2019).

There are several limitations to this study. TBSS as an analysis method has limitations in the estimation of a robust skeleton (Bach et al., 2014), especially in heterogenous regions of crossing fibers where FA fails to estimate microstructural anisotropy correctly. Moreover, it is susceptible to distortions by head movement or aberrant registrations. To guarantee the best possible data quality, several measures were performed during the analysis to ensure correct registrations of images, minimal distortion, and specificity of the TBSS skeletons for white matter tracts. Furthermore, our groups differed concerning the percentage of participants playing a musical instrument. While this means that general differences in hand motor control cannot be completely ruled out, statistical analysis of brain metrics (FA and rs-fMRI), and motor performance during the in-scanner endoscopy task (Karabanov et al., 2019) did not detect any significant between-group differences at baseline.

Taken together, this is the first study to concurrently map the effects of endoscopic surgical training on functional and structural connectivity markers in parallel and to compare these markers with task-dependent activity changes during motor skill training. We present evidence of a temporal and spatial convergence of functional and structural plasticity in the left PMv, a region that is part of the frontoparietal grasping pathway and of special importance to learning the handling of new tools. Although our findings remain explorative, they offer a platform for discussion and call for further research on the connection between functional and structural plasticity during bimanual skill learning.

## Data Availability Statement

The datasets generated for this study are available on request to the corresponding author.

## Ethics Statement

The studies involving human participants were reviewed and approved by Regional Committee on Health Research Ethics of the Capitol Region in Denmark. The participants provided their written informed consent to participate in this study. Written informed consent was obtained from the individuals for the publication of any potentially identifiable images or data included in this article.

## Author Contributions

AK, HS, KM, and TB contributed to the conception and design of the study. AK and FI acquired the data and performed data analysis. TD, KM, and KA assisted during analysis. SB conducted parts of the rs-fMRI analysis. FI wrote the first draft of the manuscript. AK, HS, TD, KA, and KM wrote sections of the manuscript. All authors contributed to the article and approved the submitted version.

## Conflict of Interest

HS has received honoraria as speaker from Sanofi Genzyme, Denmark and Novartis, Denmark, as consultant from Sanofi Genzyme, Denmark and as editor-in-chief (NeuroImage Clinical) and senior editor (NeuroImage) from Elsevier Publishers, Amsterdam, The Netherlands. He has received royalties as book editor from Springer Publishers, Stuttgart, Germany.

The remaining authors declare that the research was conducted in the absence of any commercial or financial relationships that could be construed as a potential conflict of interest.

## Footnotes

^1^http://www.surgical-science.com
