# Supplementary material for: Functional and Structural Plasticity Co-express in a Left Premotor Region During Early Bimanual Skill Learning
Source: Front Hum Neurosci. 2020 Aug 14;14:310. doi: 10.3389/fnhum.2020.00310 (PMC7456840; doi:10.3389/fnhum.2020.00310)
Supplement: Supplementary file 1 [file Table_1.DOCX]

*Supplementary Material*

**A:** Clusters of resting sensorimotor plasticity Day 0 to Day 1

| Region/ Brodmann Area (BA) | Peak MNI coordinates [x,y,z] | Cluster size (voxel) | T-value | Cluster-level P_FWE-corr_ |
| --- | --- | --- | --- | --- |
| **Left frontal lobe (total)** Precentral gyrus Postcentral gyrus  BA6 (premotor cortex)  BA4 (motor cortex) | **[-40 -8 40]** | **143**  139  28  43  1 | **4.25** | **0.029** |
| Right BA45 | [56 24 4] | 18 | 3.90 | 0.970 |
| Left BA13 | [-38 14 -6] | 19 | 3.64 | 0.964 |
| Right BA8 | [40 4 40] | 24 | 3.63 | 0.918 |
| Left BA54 | [-20 -8 -26] | 11 | 3.44 | 0.996 |
